# Supplementary figures and images for: Improving rigor and reproducibility in western blot experiments with the blotRig analysis
Source: Sci Rep. 2024 Sep 17;14:21644. doi: 10.1038/s41598-024-70096-0 (PMC11405887; doi:10.1038/s41598-024-70096-0)

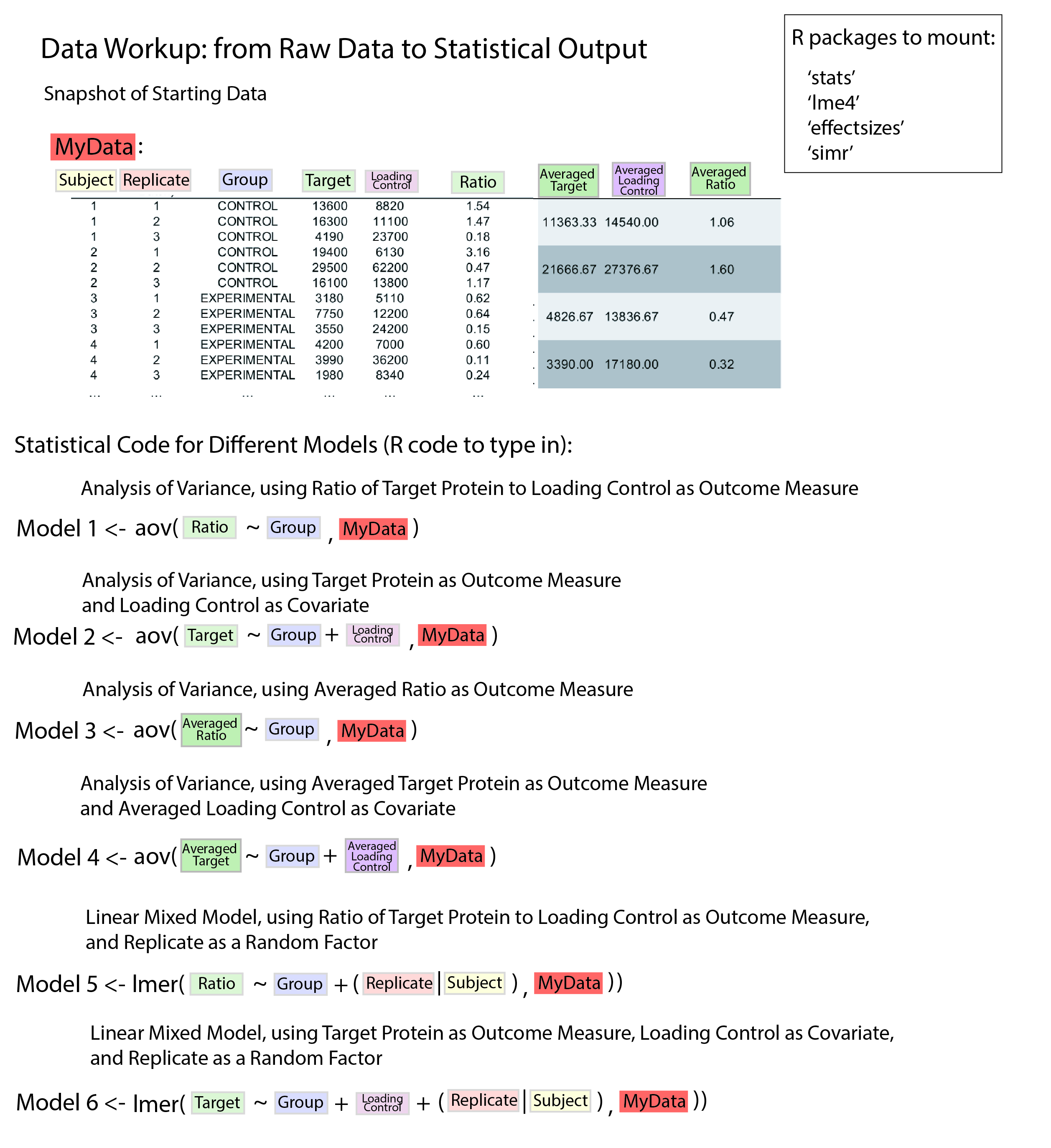

Supplement: Supplementary file 1 — Supplementary Figure 1. [file 41598_2024_70096_MOESM1_ESM.tif]

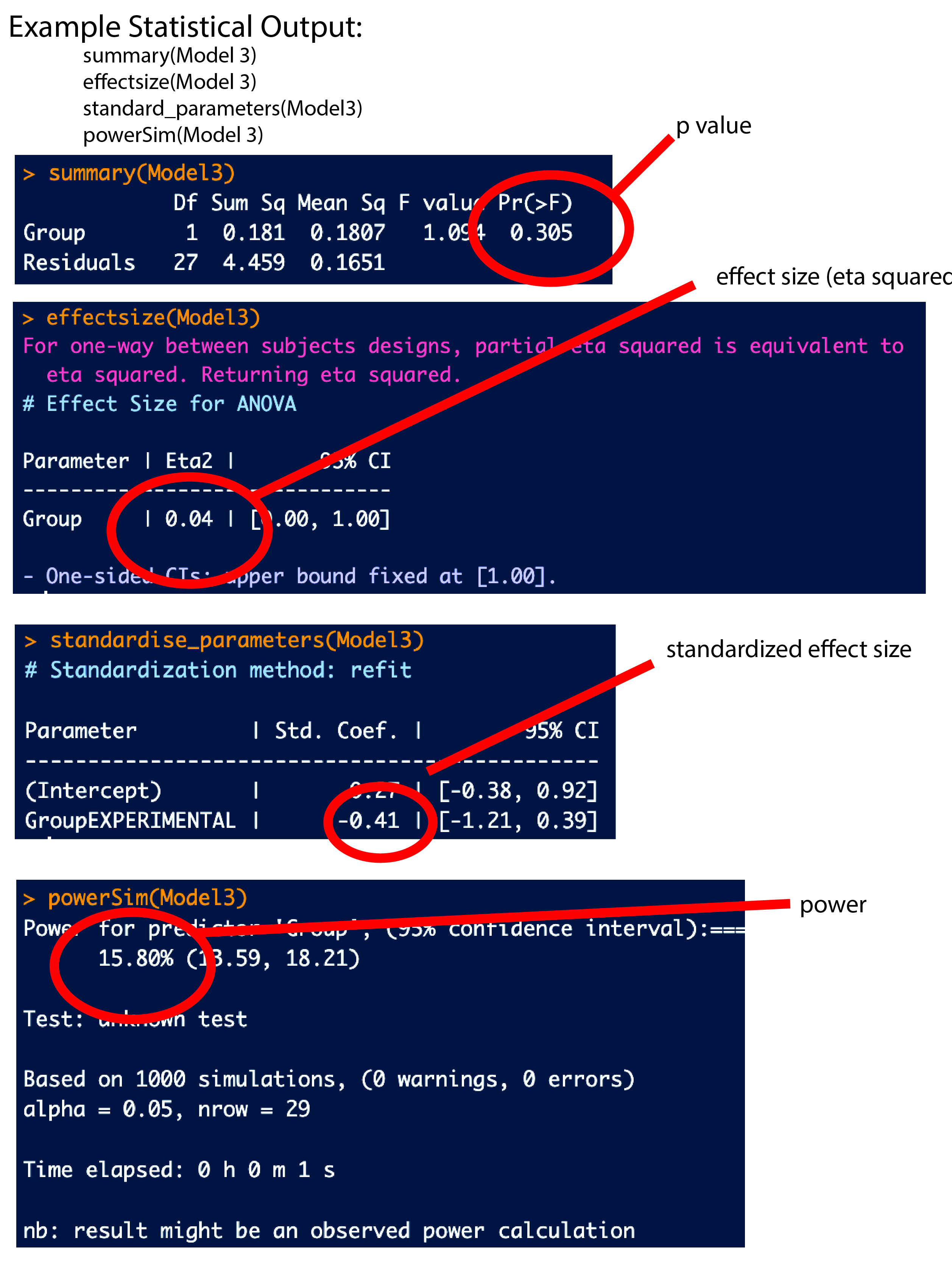

Supplement: Supplementary file 2 — Supplementary Figure 2. [file 41598_2024_70096_MOESM2_ESM.tif]
